# A Common Missense Variant in the ATP Receptor P2X7 Is Associated with Reduced Risk of Cardiovascular Events

**DOI:** 10.1371/journal.pone.0037491

**Published:** 2012-05-25

**Authors:** Olof Gidlöf, J. Gustav Smith, Olle Melander, Håkan Lövkvist, Bo Hedblad, Gunnar Engström, Peter Nilsson, Joyce Carlson, Göran Berglund, Sandra Olsson, Katarina Jood, Christina Jern, Bo Norrving, Arne Lindgren, David Erlinge

**Affiliations:** 1 Department of Clinical Sciences, Lund University, Lund, Sweden; 2 Department of Laboratory Sciences, Lund University, Lund, Sweden; 3 Department of Clinical Sciences Malmö, Lund University, Lund, Sweden; 4 Department of Neurology, Skåne University Hospital Lund, Sweden; 5 Institute of Neuroscience and Physiology, Department of Clinical Neuroscience and Rehabilitation, The Sahlgrenska Academy at University of Gothenburg, Gothenburg, Sweden; FuWai Hospital - Chinese Academy of Medical Sciences, China

## Abstract

**Background and Purpose:**

Extracellular adenosine triphosphate (ATP) regulates inflammatory cells by activation of the P2X_7_ receptor. We hypothesized that polymorphisms in *P2RX7* influence the risk of ischemic heart disease (IHD), ischemic stroke (IS) and cardiovascular risk factors and tested this hypothesis using genetic association studies.

**Methods:**

Two loss-of-function SNPs in *P2RX7* were genotyped in 1244 IHD cases and 2488 controls as well as 5969 individuals with cardiovascular risk factors. Eleven SNPs in a 250 kb region on chromosome 12 spanning *P2RX7* as well as neighboring genes *OASL*, *P2RX4* and *CAMKK2* were genotyped in 4138 individuals with IS and 2528 controls. Association was examined using linear and logistic regression models with an additive genetic model.

**Results:**

The common loss-of-function variant rs3751143 was significantly associated with a decreased risk of IHD in smokers (*P* = 0.03) as well as decreased risk of IS (OR 0.89; 95% CI = 0.81–0.97; *P* = 0.012). In addition, an intronic SNP in *CAMKK2*, rs2686342, were associated with a decreased risk of IS (OR 0.89; 95% CI = 0.82–0.97; *P* = 0.011). In subgroup analyses, both SNPs were associated with decreased risk of IS in individuals with hypertension (*P* = 0.045 and 0.015, respectively).

**Conclusions:**

A common loss-of-function missense variant in the gene encoding the P2X_7_ receptor is associated with reduced risk of IS and with IHD in smokers. These findings might implicate a role of purinergic signaling in atherogenesis or atherothrombosis.

## Introduction

Ischemic heart disease (IHD) and ischemic stroke (IS) are leading causes of death and disability in developed countries and have rapidly increasing disease rates in developing countries. A substantial proportion of cases are considered to be caused by erosion or rupture of an atherosclerotic plaque followed by thrombus formation but many risk factors, both environmental and genetic, contribute to the pathogenesis [1].

Extracellular purines and their purinergic receptors are important cardiovascular regulators and could influence the risk of cardiovascular disease by several different mechanisms including regulation of platelet aggregation, inflammation, vascular and cardiac function [2], [3]. So far, ADP receptor P2Y_12_ antagonists have achieved clinical utility in prevention of IHD and IS, [4] but several other P2 receptors constitute potential drug targets. One of the most interesting candidates is the P2X_7_ receptor. The P2X_7_ receptor induces mitosis and prevents apoptosis of T-lymphocytes and is an important activator of macrophages [5], [6]. Furthermore, it regulates the release of cytokines including interleukin-1β [7], tumour necrosis factor [8] and L-selectin, an adhesion molecule important for lymphocyte binding to endothelium [9]. All of these effects have been shown to be involved in atherogenesis.

The P2X_7_ receptor gene (*P2RX7*) is highly polymorphic. At least 11 non-synonymous polymorphisms have been identified in the coding region [10] and several of these results in altered function of the receptor. We hypothesized a role of the P2X_7_ receptor in cardiovascular disease and tested the hypothesis using a genetic association study. Findings from genetic association studies with small sample sizes frequently suffer from non-reproducibility [11]. We therefore used a large sample, the population-based Malmö Diet and Cancer study (MDCS, n = 28 449) which includes two subsamples; nested case-control samples of individuals who developed IHD (1244 cases and 2488 controls) during follow-up and a randomly selected subsample, the cardiovascular cohort (MDC-CC, n = 5969) [12] in which cardiovascular biomarkers and ultrasound measures of carotid intima-media thickness (IMT) were measured. In a secondary analysis we wanted to confirm the cardiovascular effect in a stroke sample. Association with IS was examined using three case-control studies of ischemic stroke, the MDCS (888 cases and 893 controls), the Lund Stroke Register (LSR, 2397 cases and 960 controls) and the Sahlgrenska Academy Study on Ischemic Stroke (SAHLSIS, 844 cases and 688 controls) from Southwestern Sweden.

## Methods

### Study samples

Descriptions of all samples are shown in Table 1. Sample collection, definitions and characteristics for MDCS, LSR and SAHLSIS have been described previously [12]–[15]. Briefly, MDCS is a population-based, prospective cohort study, which included 28 449 randomly selected men (born between 1926–1945) and women (born between 1923–1950) from the city of Malmö at baseline examinations between 1991 and 1996. Blood samples and information on risk factors were ascertained. Individuals who developed IHD or IS during follow-up were included in the present study together with two age and sex matched controls per case for IHD and one control per case for IS. Individuals with previous IHD or IS at baseline were excluded. A randomly selected subcohort of the MDCS, the cardiovascular cohort (MDC-CC, n = 6103), underwent B-mode ultrasound measurement of carotid intima-media thickness (IMT) and occurrence of plaques and sampling of peripheral venous blood on which plasma biomarkers including high-sensitivity C-reactive protein (hsCRP) and blood lipids were measured. From this sample, DNA was available for 5969 individuals. LSR is a prospective register study which consecutively includes all patients with a first case of ischemic or hemorrhagic stroke in the catchment area of Lund University Hospital. Blood samples and information on risk factors were collected from patients with ischemic stroke between 2001 and 2006. Control subjects were randomly selected from the same region matched by age and sex to LSR cases using the Swedish Population Register. SAHLSIS is a case-control study comprising patients with first-ever or recurrent acute IS before the age of 70 years who were enrolled between 1998 and 2008 at four stroke units in Western Sweden. Controls without cardiovascular disease were randomly selected from the same geographical region as the patients.

**Table 1:** Sample characteristics.

	Malmö Diet and Cancer-Cardiovascular Cohort (MDC-CC)	Malmö Diet and Cancer,, IHD (MDC-IHD)		Malmö Diet and Cancer, IS (MDC-IS)		Lund Stroke Register (LSR)		Sahlgrenska Academy Study on Ischemic Stroke (SAHLSIS)	
		Cases	Controls	Cases	Controls	Cases	Controls	Cases	Controls
n	5969	1244	2488	897	900	2397	960	844	668
Age	58 (5.9)	62 (6.5)	63 (6.5)	63 (6.6)	63 (6.6)	74 (12.7)	74 (12.0)	56 (10.6)	56 (10.5)
Male	42	74	74	55	54	52	57	66	59
Diabetes	9	11	4	10	3	24	7	18	5
Hypertension	64	87	71	74	58	64	47	58	34
Current smoking	28	34	27	33	21	19	10	38	20

Shown are sample characteristics with mean and standard deviation for age in years, percent male for sex and percent exposed individuals for diabetes, hypertension and current smoking.

Diabetes mellitus was defined as a physician's diagnosis of diabetes or use of antidiabetic medications in all samples except MDC-CC where fasting blood glucose was measured and values ≥6.1 mmol/l were also considered as diabetes. Blood pressure was measured using a mercury-column sphygmomanometer after 10 minutes of rest in the supine position in MDCS and SAHLSIS and ascertained from patient records in LSR. Hypertension was defined as blood pressure ≥160/90. Current smoking was ascertained from a questionnaire. Informed consent was obtained from all participants and the study was approved either by the Ethics Committee of the University of Gothenburg or by the Ethics Committee of Lund University.

### Clinical outcomes

Cardiovascular events were identified in MDCS by linkage of Swedish personal identification numbers to the Swedish Hospital Discharge Register and the Swedish Cause of Death Register for IHD and to the Stroke Register of Malmö (STROMA) [16] for IS, including events until December 31, 2003. IHD was defined as codes 410 and I21 in the *International Classification of Diseases* 8^th^, 9^th^ and 10^th^ Revisions, respectively. Ischemic stroke was ascertained in accordance with WHO criteria [17] in all samples as previously described [13], [15], [18].

### Measurement of carotid IMT and hsCRP

Common carotid IMT and IMT in the carotid bifurcation was measured using B-mode ultrasonography (Acuson 128 CT system) according to a standardized protocol by trained, certified sonographists as previously described [19]. hsCRP was measured using a high-sensitivity assay (Tina-quant, Roche Diagnostics, Basel, Switzerland) on an ADVIA 1650 Chemistry System (Bayer Healthcare, Leverkusen, Germany) with fasting plasma samples.

### Genotyping and quality control of SNPs

**Figure 1:**
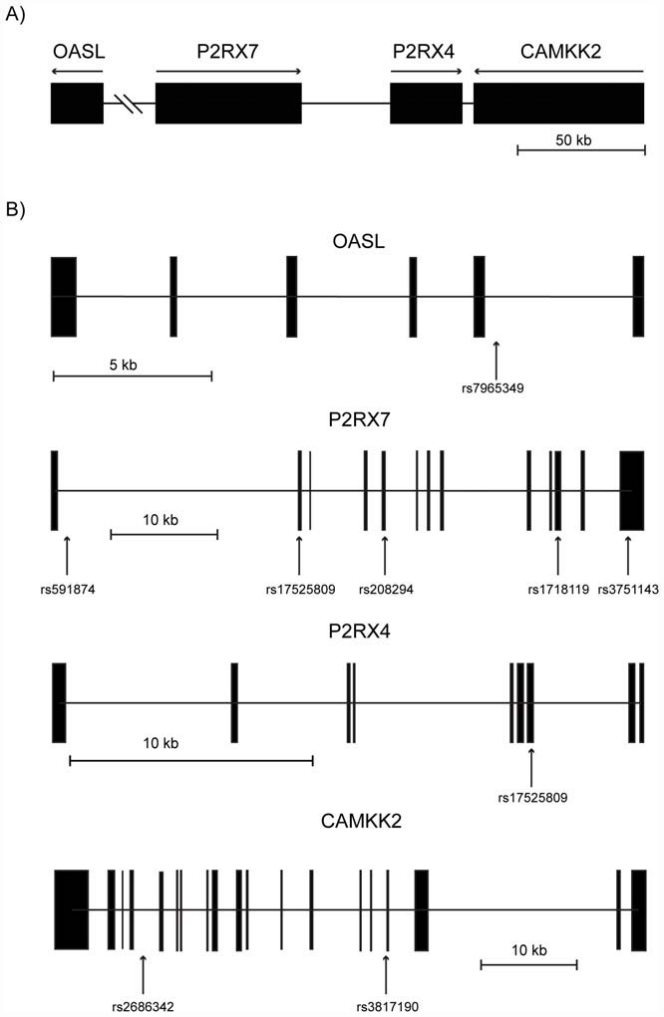
Schematic map of the genomic region containing *OASL*, *P2RX7*, *P2RX4* and *CAMKK2*. A) Filled boxes represent genes. Arrows indicate transcriptional direction. B) Filled boxes indicate exonic regions. The position of each SNP included in the study is indicated by vertical arrows.

Two loss-of-function SNPs in *P2RX7*, rs3751143 and rs2230911 were genotyped in the MDC-IHD sample. In the larger stroke sample, a total of 11 SNPs in a 250 kb region spanning *P2RX7* as well as neighboring genes *OASL*, *CAMKK2* and *P2RX4* were genotyped. A schematic map of the genomic region is shown in Figure 1. SNPs were chosen based on reported functional effects or disease associations. For LSR samples, DNA-isolation and genotyping were performed at the SWEGENE Resource Center for Profiling Polygenic Disease, later to become the Region Skåne Competence Center (RSKC) at Malmö University Hospital, Malmö, Sweden. Genotyping was performed on a MALDI-TOF mass spectrometer (SEQUENOM Mass Array) using Sequenom reagents and protocols with 10 ng DNA template. The same set of reagents was used in all samples for stroke. Automatic allele calls by SEQUENOM software were validated by manual evaluation. MDC-CC samples were genotyped using a TaqMan ABI 7900 HT according to the manufacturer's protocol with SNP genotyping assays C_15853705_20 and C_27495274_10 from Applied Biosystems. All laboratory analyses were blinded.

### Statistical analysis

Assuming an additive inheritance model, we used logistic regression analysis to test for difference of minor allele dosage between cases (IHD or IS) and controls and to estimate odds ratios for qualitative traits. SAHLSIS, LSR and MDCS were pooled for analysis of IS. For quantitative traits (i.e. intima media thickness and hsCRP), we used linear regression models with dosage of minor alleles as independent variable. hsCRP was positively skewed and was log-transformed before analysis. Adjustments were made for age, sex and cardiovascular risk factors (hypertension, diabetes and current smoking). Subgroup analyses were performed in individuals affected by known risk factors of cardiovascular disease, i.e. diabetes, hypertension and current smoking as well as in individuals with early disease onset (<60 years). P-values <0.05 were considered significant. All statistical analyses were performed in SPSS (SPSS v16, SPSS Inc., Chicago, Illinois). Hardy-Weinberg equilibrium was examined in Haploview 4.2 in each sample. Analysis of correlation between SNPs as well as calculation and visualization of linkage disequilibrium (LD) was done in Haploview 4.2 using the LSR sample. Haplotype blocks were defined using confidence interval bounds as described previously [20].

## Results

### Allele frequencies and quality control

Information on all SNPs included in the study is summarized in Table 2 and their positions in the genome are shown in Figure 1. All SNPs were successfully genotyped with <5% missing genotypes in all samples and with the exception of rs3817190 (*P* = 0.029) did not differ significantly from Hardy-Weinberg Equilibrium for cases or controls. Minor allele frequencies (MAF) were similar across samples and comparable to previous reports in the literature [10] and the HapMap project. Genotype distributions are summarized in Table S1.

**Table 2:** Description of SNPs included in the study.

SNP ID	Position, chr 12*	Position, gene	Function	% Genotyped	Alleles (major∶minor)	MAF	HWE Controls (*P*)	Samples genotyped
rs7965349	119956314	*OASL*. Intron 1	Intronic	99.5	G∶A	0.20	0.44	SAHLSIS, LSR, MDC-IS
rs591874	120055848	*P2RX7*. Intron 1	Intronic	98.5	A∶C	0.26	0.89	SAHLSIS, LSR, MDC-IS
rs17525809	120077072	*P2RX7*. Exon 2	Val76Ala	99.8	T∶C	0.06	0.52	SAHLSIS, LSR, MDC-IS
rs208294	120084636	*P2RX7*. Exon 5	Tyr155His	99.5	C∶T	0.44	0.90	SAHLSIS, LSR, MDC-IS
rs1718119	120099486	*P2RX7*. Exon 11	Ala348Thr	98.6	G∶A	0.39	0.38	SAHLSIS, LSR, MDC-IS
rs3751143	120106687	*P2RX7.* Exon 13	Glu496Ala	99.8	A∶C	0.16	0.34	SAHLSIS, LSR, MDC-IS, MDC-IHD
rs25644	120151029	*P2RX4*. Exon 7	Gly242Ser	98.6	A∶G	0.12	0.19	SAHLSIS, LSR, MDC-IS,
rs2686342	120169571	*CAMKK2*. Intron 15	Intronic	98.3	T∶A	0.20	0.81	SAHLSIS, LSR, MDC-IS,
rs3817190	120196460	*CAMKK2*. Exon 4	Ser85Thr	98.4	A∶T	0.39	0.03	SAHLSIS, LSR, MDC-IS,
rs2230911	120099514	*P2RX7.* Exon 11	Thr357Ser	95.4	A∶G	0.17	0.40	LSR, MDC-IHD
rs2230912	120106579	*P2RX7*. Exon 13	Arg460Glu	95.7	C∶G	0.09	1	LSR

* Reference sequence NT_009775.16.

MAF∶minor allele frequency, HWE: Hardy-Weinberg equilibrium.

### Genotypic association with ischemic heart disease

In the first stage of the investigation, two loss-of-function SNPs in *P2RX7*, rs3751143 and rs2230911 were genotyped in the MDC-IHD sample. Neither of the SNPs were significantly associated with IHD (p>0.05). In secondary analyses, the minor allele of rs3751143 was protective of IHD in smokers (OR = 0.77; 95% CI = 0.61–0.97; P = 0.03, n = 1091) but not in non-smokers (OR = 1.05; 95% CI = 0.90–1.23; P = 0.56, n = 2638). The association improved slightly after adjustment for age and sex (OR = 0.76; 95% CI = 0.60–0.96; P = 0.021). In smokers, the risk of IHD was lower in homozygotes for the minor allele (n = 9 cases and 29 controls) than in homozygotes for the major allele (n = 306 cases and 430 controls) with an odds ratio of 0.44 (95% CI = 0.20–0.93; p = 0.03). The risk for heterozygotes with current smoking (n = 103 cases and 173 controls) was not significantly different from homozygotes for the minor allele, C (OR = 0.84; 95% CI = 0.63–1.11; p = 0.22).

### Intima-media thickness and hsCRP

Neither of the SNPs analyzed in MDC-CC were significantly associated with common or internal carotid IMT, hsCRP, blood pressure, blood lipids, body-mass index or diabetes (p>0.05).

### Genotypic association with ischemic stroke

To confirm the cardiovascular effect of rs3751143 and to elucidate whether a correlated SNP might explain the association in MDC-IHD, a total of 11 SNPs were genotyped in the three stroke samples. In addition to the two loss-of-function SNPs in *P2RX7* genotyped in MDC-IHD, nine SNPs in *P2RX7* as well as neighboring genes *OASL*, *CAMKK2* and *P2RX4* were analyzed. The additional SNPs were chosen based on functional effects or relevant disease associations described in the literature [10], [21]–[29]. IS was significantly associated with rs3751143 (*P2RX7*c. 1488A>C, p. Glu496Ala) and rs2686342 (*CAMKK2*c. 1452+1221A>T). The minor alleles of both SNPs were associated with a decreased risk of developing IS with an OR of 0.89 (95% CI = 0.81–0.97, *P* = 0.012) for rs3751143 and 0.89 (95% CI = 0.82–0.97, *P* = 0.011) for rs2686342. The associations were not affected substantially by adjustment for cardiovascular risk factors (OR = 0.87; 95% CI = 0.79–0.97; *P* = 0.008 and OR = 0.88; 95% CI = 0.80–0.97; *P* = 0.009, respectively). Odds ratios for rs3751143 in each individual sample were 0.88 (95% CI = 0.76–1.02) in LSR, 0.84 (95% CI = 0.70–1.01) in MDC-IS and 0.97 (95% CI = 0.80–1.17) in SAHLSIS after adjustments for age and sex. Odds ratios for rs2686342 in each sample were 0.91 (95% CI = 0.79–1.04) in LSR, 0.88 (95% CI = 0.75–1.04) in MDC-IS and 0.94 (95% CI = 0.78–1.14) in SAHLSIS after adjustments for age and sex. Adjustment for cardiovascular risk factors did not substantially affect the odds ratios or confidence intervals in either study (Data not shown). In subgroup analyses, both rs3751143 and rs2686342 minor alleles were significantly associated with decreased IS risk in individuals with hypertension (n = 3889; OR = 0.88; 95% CI = 0.77–1.00; *P* = 0.045 and OR = 0.86; 95% CI = 0.76–0.97; *P* = 0.015, respectively) but not in non-hypertensive individuals (n = 2703; OR = 0.88; 95% CI = 0.76–1.02; *P* = 0.06 and OR = 0.92; 95% CI = 0.81–1.06; *P* = 0.26, respectively). The minor allele of rs2686342 was also associated with a more pronounced risk reduction in individuals with diabetes (n = 940; OR = 0.70; 95% CI = 0.51–0.97; *P* = 0.030) than without diabetes (n = 5531; OR = 0.90; 95% CI = 0.82–0.99; *P* = 0.034). Neither SNP was significantly associated with IS in patients with early disease onset or current smoking even though effect estimates were similar but with wider confidence intervals.

Pair-wise correlations between each of the two SNPs significantly associated with IS and the rest of the SNPs in the study were analyzed in the LSR sample (Table S2). rs3751143 and rs2686342 were more strongly correlated with each other (r^2^ = 0.24) than with any other SNP included in the study.

### Testing for LD

**Figure 2:**
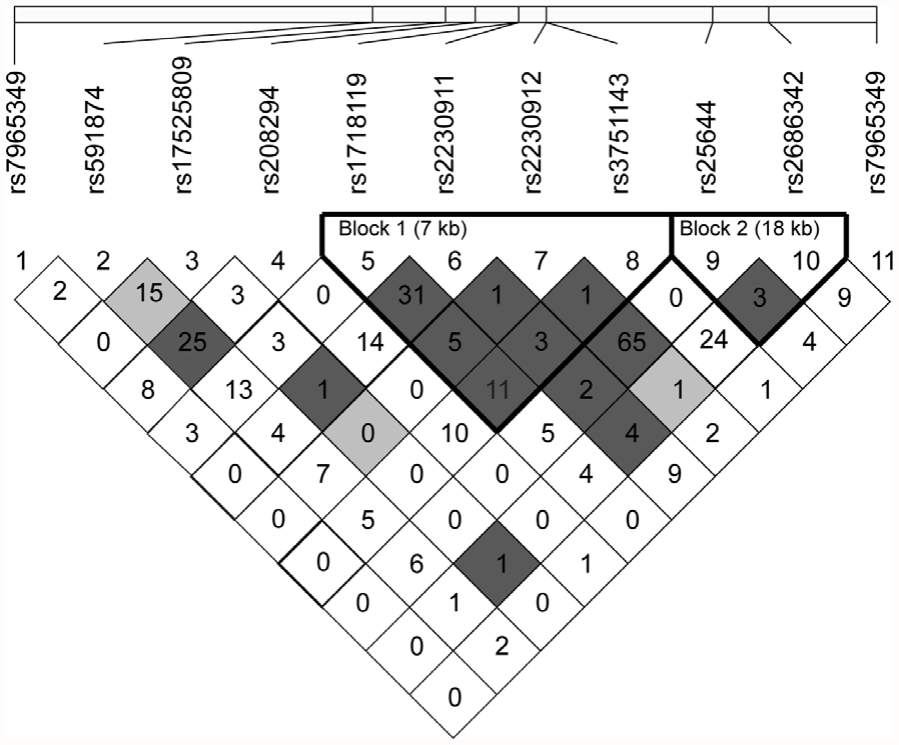
Patterns of linkage disequilibrium within *P2RX7*, *OASL*, *CAMKK2* and *P2RX4*. Shown are r^2^-values between polymorphic loci in the LSR sample. SNPs lying within blocks are depicted in bold type. Dark grey background: strong linkage; light grey background: uninformative; white background: recombination.

We examined the patterns of LD between alleles at polymorphic loci using all SNPs in the LSR sample (Figure 2). We detected two independent haplotype blocks within the investigated region. The first block is approximately 7 kb long and spans four SNPs in exon 11 and 13 of *P2RX7*. The second block is 18,5 kb in size and spans two SNPs, one in exon 7 of *P2RX4* and one in intron 3 of *CAMKK2*. The SNPs that were significantly associated with IS (rs3751143 and rs2686342) were located on separate blocks.

## Discussion

In this large genetic association study we detected that a loss of function variant of the pro-inflammatory ATP receptor P2X7 was associated with reduced risk of ischemic heart disease in the subgroup of smokers. Together with pre-clinical data this indicates a possible role in atherosclerotic disease. We therefore examined the variant and neighbouring SNPs in a large case-control stroke population. Here we found that the variant was associated with reduced risk of ischemic stroke.

We found that the minor allele of rs3751143 confers a reduced risk of ischemic heart disease in smokers. This association constitutes a subgroup analysis and hence should be considered with caution. However, known loss of function in the genetic variant [29] and the corresponding effect detected in IS makes our finding more likely to constitute a ‘true’ association. Increased levels of systemic inflammation have been described in smokers and may be associated both to an increased atherosclerotic burden and to chronic inflammation, often manifesting as chronic obstructive pulmonary disease (COPD). It is therefore conceivable that an anti-inflammatory polymorphism might be of greater benefit in smokers.

When examined in a large stroke population we could confirm the cardiovascular effect of rs3751143. The minor alleles of rs3751143, as well as of a SNP in neighboring gene *CAMMK2*, rs2686342, were associated with a decreased risk of ischemic stroke. rs3751143 is located in exon 13 of *P2XR7* and is particularly interesting since it confers a total loss of function of the P2X7 receptor [29] (Glu496Ala). It has been associated with a reduced clearance of *M. tuberculosis* by macrophages, increased susceptibility to extra pulmonary tuberculosis and decreased efficiency in killing intracellular Toxoplasma gondii [10], [21], [28]. Furthermore, a recent study showed that P2X7 receptor deficient mice were protected from thrombosis in vivo [30]. rs2686342 is located in intron 8 of *CAMKK2* and little is known about its functional effects. However, *CAMKK2*-deficient mice have been shown to be protected against inflammation [31]. LD analysis showed that the two SNPs were located on separate haplotype blocks and linkage is therefore unlikely.

For the association study of ischemic stroke we used three samples to increase our sample size. This sample size should allow adequate statistical power to detect a modest risk estimate, based on previous power analyses [32]. All samples were from southwestern Sweden and have a uniform population history. It is therefore unlikely that population stratification had a significant impact on our findings as discussed previously [14]. The effect of P2X_7_R Ala496 (rs3751143) in the SAHLSIS sample differed somewhat from that in LSR and MDC but the confidence interval overlaps with the effects in the other samples. The observations of similar effects for Ala496 (rs3751143) in all stroke samples separately with improved association with pooling and the significant association in subgroups at high risk makes the association more likely to be true.

Atherosclerosis is now considered an inflammatory disease in which the macrophage is recruited to the cholesterol rich plaque and activated by oxidized LDL [1]. The ATP-mediated activation of the P2X_7_ receptor has been shown to be important for macrophage activation. Our results suggest that loss of receptor function is also associated with lower risk of cardiovascular disease. If our findings can be replicated in additional samples and the mechanism verified in functional analyses, this could motivate inhibitors of P2X_7_ as the second antipurinergic treatment to reach clinical trials for cardiovascular disease.

## Supporting Information

Table S1 **Genotype distributions of SNPs analyzed in the stroke samples and in the ischemic heart disease sample.** **A.** Genotype distributions of SNPs analyzed in the stroke samples. **B.** Genotype distributions of SNPs analyzed in the ischemic heart disease sample.
(DOCX)

Table S2 **Pair-wise correlations of rs3751143 and rs2686342 and the other SNPs in the LSR sample.** **A.** Pair-wise correlations of rs3751143 and the other SNPs in the LSR sample. **B.** Pair-wise correlations of rs2686342 and the other SNPs in the LSR sample.
(DOCX)
