# Supplementary material for: A Common Missense Variant in the ATP Receptor P2X7 Is Associated with Reduced Risk of Cardiovascular Events
Source: PLoS One. 2012 May 25;7(5):e37491. doi: 10.1371/journal.pone.0037491 (PMC3360776; doi:10.1371/journal.pone.0037491)
Supplement: Table S1 — Genotype distributions of SNPs analyzed in the stroke samples and in the ischemic heart disease sample. A. Genotype distributions of SNPs analyzed in the stroke samples. B. Genotype distributions of SNPs analyzed in the ischemic heart disease sample. (DOCX) [file pone.0037491.s001.docx]

**Table S1a.** Genotype distributions of SNPs analyzed in the stroke samples

|  | Cases | Controls |
| --- | --- | --- |
| rs25644 |  |  |
| AA | 3149 (77,7) | 1981 (79,8) |
| AG | 847 (20,9) | 463 (18,7) |
| GG | 59 (1,5) | 37 (1,5) |
|  |  |  |
| rs208294 |  |  |
| CC | 1303 (32,1) | 764 (31,0) |
| TC | 1988 (48,9) | 1210 (49,2) |
| TT | 774 (19,0) | 487 (19,8) |
|  |  |  |
| rs591874 |  |  |
| AA | 2219 (54,7) | 1401 (56,6) |
| CA | 1550 (38,2) | 923 (37,3) |
| CC | 284 (7,0) | 153 (6,2) |
|  |  |  |
| rs1718119 |  |  |
| AA | 637 (15,7) | 380 (15,3) |
| GA | 1889 (46,6) | 1157 (46,7) |
| GG | 1529 (37,7) | 943 (38,0) |
|  |  |  |
| rs2686342 |  |  |
| AA | 146 (3,6) | 105 (4,3) |
| AT | 1263 (31,1) | 821 (33,4) |
| TT | 2648 (65,3) | 1533 (62,3) |
|  |  |  |
| rs3751143 |  |  |
| AA | 2894 (70,9) | 1668 (67,6) |
| CA | 1090 (26,7) | 730 (29,6) |
| CC | 98 (2,4) | 69 (2,8) |
|  |  |  |
| rs3817190 |  |  |
| AA | 1443 (35,8) | 901 (37,2) |
| AT | 1967 (48,8) | 1110 (45,8) |
| TT | 620 (15,4) | 412 (17,0) |
|  |  |  |
| rs7965349 |  |  |
| AA | 162 (4,0) | 110 (4,5) |
| GA | 1322 (32,4) | 788 (32,1) |
| GG | 2590 (63,6) | 1557 (63,4) |
|  |  |  |
| rs17525809 |  |  |
| CC | 11 (0,3) | 7 (0,3) |
| CT | 480 (11,8) | 291 (11,8) |
| TT | 3592 (88,0) | 2167 (87,9) |
|  |  |  |
| rs2230911* |  |  |
| AA | 1226 (70,0) | 625 (69,1) |
| AG | 474 (27,0) | 259 (28,6) |
| GG | 52 (3,0) | 21 (2,3) |
|  |  |  |
| rs2230912* |  |  |
| CC | 1446 (82,6) | 777 (85,0) |
| CG | 289 (16,5) | 132 (14,4) |
| GG | 52 (3,0) | 5 (0,6) |

**Table S1b.** Genotype distributions of SNPs analyzed in the ischemic heart disease sample

|  | Cases | Controls |
| --- | --- | --- |
| rs3751143 |  |  |
| AA | 845 (69,6) | 1648 (68,6) |
| CA | 336 (27,7) | 680 (28,3) |
| CC | 33 (2,7) | 76 (3,2) |
|  |  |  |
| rs2230911 |  |  |
| AA | 995 (83,4) | 1976 (84,0) |
| AG | 190 (15,9) | 359 (15,3) |
| GG | 8 (0,7) | 17 (0,7) |

Genotype distributions are shown for cases and controls with numbers and percentages within parentheses. *Only genotyped in the LSR sample
