# Supplementary material for: A Common Missense Variant in the ATP Receptor P2X7 Is Associated with Reduced Risk of Cardiovascular Events
Source: PLoS One. 2012 May 25;7(5):e37491. doi: 10.1371/journal.pone.0037491 (PMC3360776; doi:10.1371/journal.pone.0037491)
Supplement: Table S2 — Pair-wise correlations of rs3751143 and rs2686342 and the other SNPs in the LSR sample. A. Pair-wise correlations of rs3751143 and the other SNPs in the LSR sample. B. Pair-wise correlations of rs2686342 and the other SNPs in the LSR sample. (DOCX) [file pone.0037491.s002.docx]

**Table S2a.** Pair-wise correlations of rs3751143 and the other SNPs in the LSR sample

|  | r^2^ |
| --- | --- |
| rs7965349 | 0 |
| rs591874 | 0,05 |
| rs17525809 | 0 |
| rs208294 | 0,1 |
| rs1718119 | 0,11 |
| rs2230911 | 0,03 |
| rs2230912 | 0,01 |
| rs25644 | 0 |
| rs2686342 | 0,24 |
| rs3817190 | 0,01 |

**Table S2b.** Pair-wise correlations of rs2686342 and the other SNPs in the LSR sample

|  | r2 |
| --- | --- |
| rs7965349 | 0 |
| rs591874 | 0,01 |
| rs17525809 | 0,01 |
| rs208294 | 0 |
| rs1718119 | 0,04 |
| rs2230911 | 0,04 |
| rs2230912 | 0,01 |
| rs3751143 | 0,24 |
| rs25644 | 0,03 |
| rs3817190 | 0,04 |
